# Functional Profiling of Precursor MicroRNAs Identifies MicroRNAs Essential for Glioma Proliferation

**DOI:** 10.1371/journal.pone.0060930

**Published:** 2013-04-05

**Authors:** Saija Haapa-Paananen, Ping Chen, Kirsi Hellström, Pekka Kohonen, Sampsa Hautaniemi, Olli Kallioniemi, Merja Perälä

**Affiliations:** 1 Medical Biotechnology, VTT Technical Research Centre of Finland, Turku, Finland; 2 Computational Systems Biology Laboratory, Institute of Biomedicine and Genome-Scale Biology Research Program, University of Helsinki, Helsinki, Finland; 3 FIMM - Institute for Molecular Medicine Finland, University of Helsinki, Helsinki, Finland; Columbia University, United States of America

## Abstract

Cancer initiation and progression involve microRNAs that can function like tumor suppressors and oncogenes. The functional significance of most miRNAs is currently unknown. To determine systematically which microRNAs are essential for glioma growth, we screened a precursor microRNA library in three human glioblastoma and one astroglial cell line model systems. The most prominent and consistent cell proliferation–reducing hits were validated in secondary screening with an additional apoptosis endpoint. The functional screening data were integrated in the miRNA expression data to find underexpressed true functional tumor suppressor miRNAs. In addition, we used miRNA-target gene predictions and combined siRNA functional screening data to find the most probable miRNA-target gene pairs with a similar functional effect on proliferation. Nine novel functional miRNAs (hsa-miR-129, -136, -145, -155, -181b, -342-5p, -342-3p, -376a/b) in GBM cell lines were validated for their importance in glioma cell growth, and similar effects for six target genes (*ROCK1, RHOA, MET, CSF1R, EIF2AK1, FGF7*) of these miRNAs were shown functionally. The clinical significance of the functional hits was validated in miRNA expression data from the TCGA glioblastoma multiforme (GBM) tumor cohort. Five tumor suppressor miRNAs (hsa-miR-136, -145, -342, -129, -376a) showed significant underexpression in clinical GBM tumor samples from the TCGA GBM cohort further supporting the role of these miRNAs *in vivo*. Most importantly, higher hsa-miR-145 expression in GBM tumors yielded significantly better survival (p<0.005) in a subset of patients thus validating it as a genuine tumor suppressor miRNA. This systematic functional profiling provides important new knowledge about functionally relevant miRNAs in GBM biology and may offer new targets for treating glioma.

## Introduction

MicroRNAs (miRNAs) are about 22–25 nt long, noncoding RNAs originally discovered in *Caenorhabditis elegans* [1]. MiRNA is produced from primary transcripts via release of 60–70 nt stem-loop intermediates known as pre-miRNA, which are then cleaved by Dicer to produce double-stranded RNA duplexes consisting of mature miRNAs and their antisense strand. Cancer initiation and progression can involve miRNAs as reviewed by [2], [3]. MiRNAs can function as tumor suppressors and oncogenes or “oncomirs” [4]. Functional studies have also revealed that miRNAs play an important role in regulating various cellular processes, including proliferation, stem cell renewal, and differentiation [5].

Glioblastoma multiforme (GBM) is the most common and aggressive primary brain tumor, with approximately 10,000 new cases per year in the US [6]. Patients with newly diagnosed GBM have a median survival period of approximately one year. Similarly to other cancers, a characteristic microRNA expression pattern is seen in glioblastomas. For instance, several brain-enriched miRNAs, miR-128, miR-181a, miR-181b, and miR-181c, are mainly down-regulated in glioblastomas [7], whereas miR-221 and miR-21 are strongly up-regulated in GBM and grade II–IV astrocytic tumors [8].

Each deregulated miRNA can target hundreds or even thousands of genes by transcriptional silencing at the mRNA level or translational inhibition at the protein level. For a few miRNAs, their specific individual target genes have been proven with 3-untranslated region (3′UTR) assays, and functions have been analyzed by down-regulating or overexpressing the miRNAs. However, no genome-wide studies exist with a systematic analysis of miRNA functions in gliomas. In this study, we used miRNA precursor libraries systematically in overexpression screenings to identify miRNAs that reduce proliferation or increase apoptosis in glioma cell lines. In cancer, the loss of tumor-suppressive miRNAs enhances the expression of target oncogenes. Since each miRNA can target thousands of genes, we used target predictions and combined small interfering RNA (siRNA) functional screening data to find the most probable miRNA-target gene pairs that have similar functional effects. Six genes were found and validated at the miRNA and gene expression level in clinical GBM samples.

## Materials and Methods

### Cell Culture and Reagents

Human glioblastoma cell lines A172 and U87MG were obtained from ECACC (Salisbury, UK) in 2003, LN405 from the DSMZ (Braunschweig, Germany) in 2003, and astroglia SVGp12 from American Type Culture Collection (Manassas, VA, USA) in 2006. Cells were cultured in medium conditions recommended by the providers for less than 4 months before being used in these experiments. All siRNAs were purchased from Qiagen (Qiagen GmbH, Hilden, Germany). Additional control siRNAs were AllStars Hs Cell Death Control siRNA (Qiagen) and Negative Control siRNA (siNEG, Qiagen). The siRNAs were used at a final concentration of 15 nM each.

Total cellular RNA including small RNAs was isolated for miRNA expression profiling using the mirVana miRNA Isolation Kit (Ambion Inc., Austin, TX, USA). Total cellular RNA for gene expression profiling was extracted using TRIzol reagent (Invitrogen, Carlsbad, CA, USA) followed by purification of the RNA using Qiagen’s RNeasy columns (Valencia, CA, USA) according to the manufacturer’s instructions.

### MicroRNA Functional Screening and Data Analysis

The A172, LN405, U87MG, and SVGp12 cells were transfected with 20 nM human Pre-miR miRNA Precursor library v2 (Ambion Inc., 319 molecules). Negative controls included in the screenings were pre-miR miRNA precursor negative controls #1 and #2 (Ambion) and a scrambled control mirCURY knockdown probe (Exiqon A/S, Vedbaek, Denmark). For cell viability screening, miRNAs were printed (Hamilton Bonaduz AG, Bonaduz, Switzerland) in 384-well plates (Greiner Bio-One, Stonehouse, Germany) to reach the final assay concentration of 20 nM. SilentFect transfection agent (Bio-Rad Laboratories, Hercules, CA, USA) was diluted in OptiMEM (Gibco Invitrogen, Paisley, CA, USA), incubated for 10 min at room temperature, and aliquoted to wells using Multidrop 384 Microplate Dispenser (Thermo Labsystems, Thermo Electron Corporation, Waltham, MA, USA). Plates were then incubated for 1 h at room temperature. The predetermined optimal number of cells (1,000–1,500 cells per well) was then added to the plates, and further incubated for 72 h at 37°C with 5% CO_2_. Cell viability was measured with the CellTiterBlue assay (Promega Corp, Madison, WI, USA). The EnVision Multilabel Plate Reader (PerkinElmer Inc., Waltham, MA, USA) was used for signal quantification. The miRNA screening data were normalized plate-wise for row and column effects, and the results from the replicate screenings were combined for the analyses.

### Secondary Validation Assays for miRNA Functional Screening

Transfections were performed as described in the microRNA functional screening and data analysis section but in 6-well and 384-well formats. Cell viability was measured with the CellTiter-Glo assay, and induction of caspase-3 and -7 activities was detected with the homogenous Caspase-Glo 3/7 Assay (Promega Corp, Madison, WI, USA). The EnVision Multilabel Plate Reader (PerkinElmer) was used for signal quantification. The miRNA screening data was normalized plate-wise for row and column effects, and the results from the replicate screenings were combined for the analyses.

### Live Cell Time-lapse Microscopy

Cell growth and confluence were monitored by taking time-lapse phase contrast images with an IncuCytePlus microscope (Essen Instruments Inc, Ann Arbor, MI, USA) once per hour of live cells grown for 4 days in a CO_2_ incubator.

### Cell Cycle Assays

The cell cycle was analyzed using the Click-it Edu Alexa 647 nm fluor HCS assay kit (Invitrogen) according to the Invitrogen protocol by applying the 96-well protocol to the 384-well format and staining cells with Hoechst 33342 stain. Cells were visualized using ScanR automated fluorescence microscopy (Olympus Corp, Tokyo, Japan).

### MiRNA Expression Analyses

MiRNA expression was profiled with the Agilent Human miRNA Microarray Kit (V1) (Agilent Technologies, Santa Clara, CA, USA). The RNA was isolated from A172, LN405, U87MG, and SVGp12 cells with the mirVana miRNA Kit (Applied Biosystems) and labeled according to the Agilent protocol (version 1.0, April 2007). The arrays were scanned with the Agilent Microarray G2565 Scanner; the scanner was controlled with Agilent Scan Control software. Default settings recommended by Agilent were used as the scanning parameters (including the use of an extended dynamic range with green PMT set to XDR Hi 100% and XDR Lo 5%). Agilent Feature Extraction Software (version 9.5) was used to extract the data. Human normal brain total RNAs including miRNAs (Ambion) were used as the reference sample. The data were normalized using the vsn R-package and generalized log_2_ variance stabilization. Each column was calibrated by an affine transformation, and then all the data were transformed with a variance-stabilizing transformation [9] to remove systematic array biases and make the variances independent of the mean intensity. The resulting ratios were log_2_ scale miRNA expression in the cell line compared to normal brain reference tissue expression.

### Gene Expression Profiling of Gliomas on Oligonucleotide Microarrays

MiRNA expression was profiled at the genome-wide gene expression level using the Affymetrix U133 Plus 2.0 GeneChip (Affymetrix, Santa Clara, CA, USA) according to the manufacturer’s instructions. Before hybridization, the yield and quality of the total RNA were evaluated with the NanoDrop ND-1000 UV-Vis Spectrophotometer, and the integrity of the RNA provided was assessed using the Agilent Technologies 2100 Bioanalyzer.

### Integration of miRNA Expression and Functional Profiling Data

**Figure 1:**
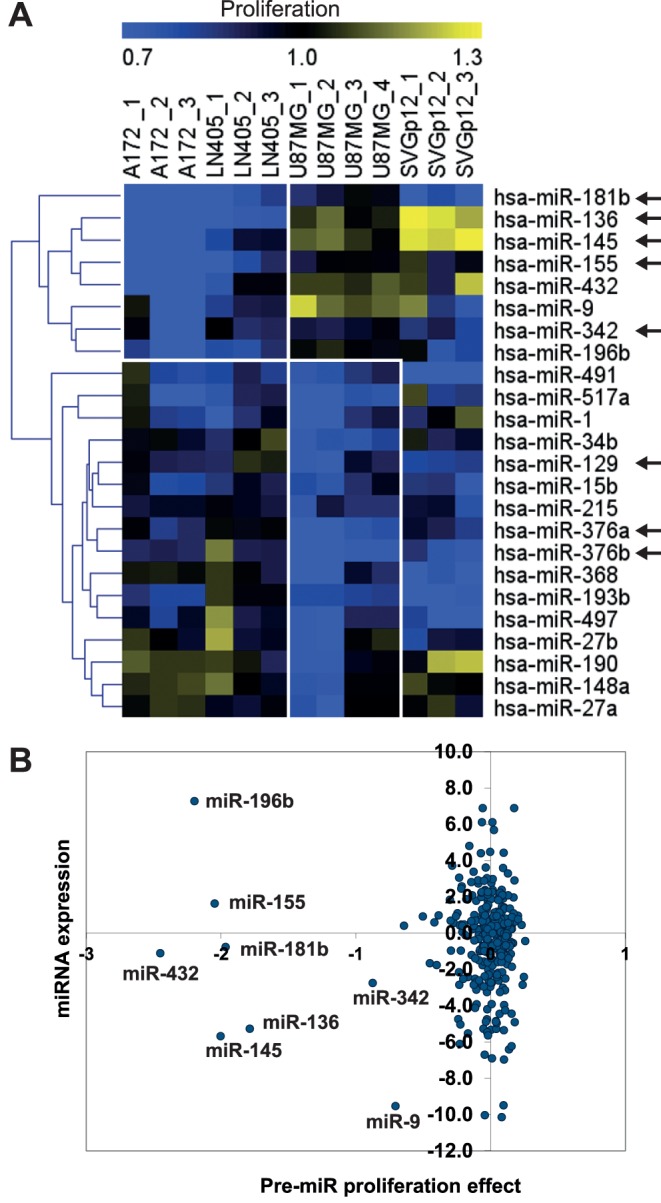
Functional screening for miRNAs affecting glioma cell line proliferation after overexpression of precursor miRNAs. A) Hierarchical clustering of repeating hits from the miRNA pre-miR functional proliferation primary screening using the Euclidean method in MeV 4.8 (row and column normalized data) [10], [11]. In the linear relative scale, 1.0 denotes no change in proliferation (100%), and 0.7 denotes 30% reduction in proliferation. Each screening was performed in three to four biological replicates. Arrows denote miRNAs that were selected for further validation. B) Integration of miRNA expression data and primary screening functional data for the A172 glioma cell line shown in a scatter-plot (log_2_ mean data on both axes). MiRNA expression is presented as the ratio of expression in the cell line compared to the normal brain reference miRNA (log_2_). Some miRNAs highlighted with arrows in panel A are marked in panel B.

To compare the miRNA expression levels to the proliferation effects caused by pre-miR transfections, we plotted the two sets of data in one scatter-plot for each cell line (Figure 1B). We used the twofold difference from the median of all cancer cell lines as a significant deviation from average miRNA expression. For the functional screening data, we set the hit cut-off to two standard deviations from the normalized mean of the screenings.

### Small Interfering RNA Primary and Secondary Screening

SiRNA screening was performed essentially the same way as the miRNA screening. Briefly, the A172, LN405, U87MG, and SVGp12 cells were transfected with the 15 nM human Kinase siRNA library (2241 siRNAs, Ambion Inc.) or human Cancer siRNA Set (2375 siRNAs, Qiagen GmbH) using Silentfect (Bio-Rad Laboratories) in a 384-well format. The predetermined optimal number of cells (1,000–1,500 cells per well) was then added to the plates, and further incubated for 72 h at 37°C with 5% CO_2_. Cell viability was measured with CellTiter-Blue or CellTiter-Glo assays, and induction of caspase-3 and -7 activities was detected with homogenous ApoOne assay (Promega). The EnVision Multilabel Plate Reader (PerkinElmer) was used to quantify the signals. The siRNA screening data were normalized plate-wise for row and column effects, and the results from the replicate screenings were combined for the analyses (only part of the data is shown).

### Computational Data Analysis and Statistical Analysis

Hierarchical clustering was performed with the TM4 Multi Experiment Viewer 4.8 [10], [11]. The microRNA target filter of the Ingenuity Pathways Analysis (IPA; Ingenuity Systems) program was used to analyze the miRNAs and their predicted targets, and to integrate the target gene expression in the glioma cell lines. Student’s t-test was used to calculate the p-values for the experiments. All p-values are two-sided with the number of samples (*n*) shown in the figure text.

TCGA GBM data of 308 patients with clinical information and 9 normal brain tissues were downloaded and analyzed by Anduril integration tool [12]. Statistical analysis, Student’s T-test and log2-scale fold change, were done on level 3 microRNA expression data from TCGA and transcript expression data preprocessed by Multiple Exon Array Preprocessing (MEAP) algorithm [13] for candidate microRNAs and their targets. Kaplan-Meier analysis was performed as described previously in Ovaska et al. [12], where patients were grouped based on the expression differences from the median expression of normal tissues (low level: -1 group; high level: 0 group).

## Results

### Systematic Functional Screening of microRNAs

To discover the miRNAs that affect cell proliferation, three glioblastoma cell lines, A172, LN405, and U87MG, and one control SV40 transformed fetal astroglial cell line, SVGp12, were screened in a 384-well format for cell proliferation using a human pre-miR miRNA Precursor library (Ambion) containing 319 miRNA precursor molecules. Cell viability was determined via adenosine triphosphate (ATP) quantification, as an indication of metabolically active cells, with the CellTiterBlue luminescence assay (Promega Corp). Primary proliferation screening data was normalized plate-wise for row and column effects, and the results from three to four replicate screenings were combined for the analyses (Figure 1A). Hits for miRNA precursors that reduce proliferation were determined as two standard deviations below the mean of the replicate wells. In total, 24 potential proliferation-reducing precursor miRNA molecules were found in the primary screening (Table S1). These miRNAs were selected for secondary validation screening with proliferation and additional apoptosis endpoint with the homogenous Caspase-Glo 3/7 Assay (Promega) (Figure S1). In total, nine miRNAs were validated in the secondary screening as being repeating proliferation-reducing and/or apoptosis-increasing hits when the cells were transfected with the pre-miR miRNA precursor molecules (Ambion Inc.) (marked with arrows in Figure 1A). For further validation, two of the cell lines (A172 and LN405) were also screened for cell proliferation and apoptosis induction using a larger precursor library with 819 molecules (Dharmacon, Lafayette, CO, USA). Eight of the nine previously identified miRNAs (hsa-miR-136, -145, -155, -181b, -342-5p, -342-3p, -376a/b) were also validated in the Dharmacon precursor miRNA screenings in the A172 and LN405 cell lines. One of the nine miRNA precursors, miR-129, had a hit only in the U87MG cell line, which was not screened with the Dharmacon precursor library.

### Integration of miRNA Expression and Functional Profiling Data

Each of the four cell lines was profiled for miRNA and mRNA expression. The data from the functional miRNA precursor screening were integrated in the miRNA expression profiling data to find how the nine miRNA precursors are expressed in the cancer cell lines compared to normal brain reference RNA. Figure 1B shows the miRNA expression of the A172 glioblastoma plotted against the precursor miRNA proliferation effects from the primary screenings. Six miRNAs (miR-181b, -432, -136, -145, -342, -9) cluster clearly separately based on the stronger effect on proliferation of the precursor miRNAs and are underexpressed in the A172 cell line compared to the normal brain (Figure 1B). The precursor miR-196b and miR-155 show an effect on proliferation but are overexpressed. The proliferation effects of miR-9, miR-196b, and miR-432 were not consistent in all cell lines and were therefore left out from further validation.

### Functional Validation of the Selected miRNAs

**Figure 2:**
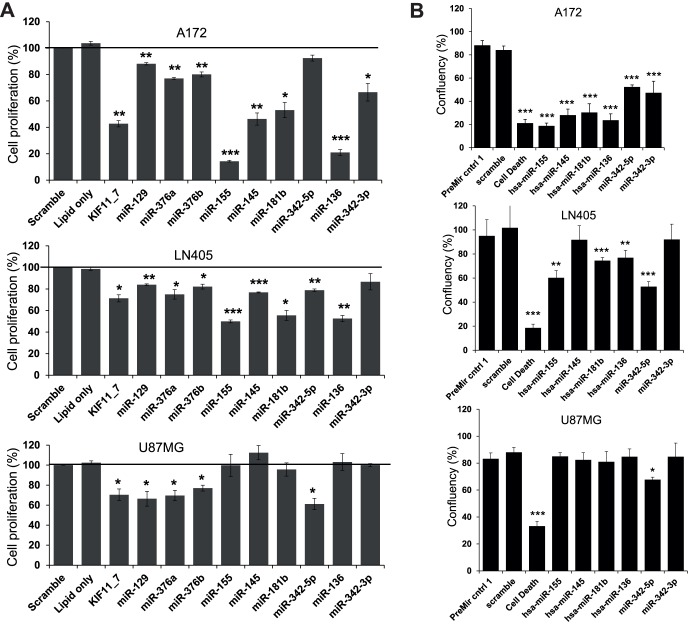
Validation of hit precursor miRNAs that reduced proliferation with A) CellTiter-Glo assay at 72 h after transfection (n = 3 biological, 4 technical repeats), B) monitoring cell growth and confluence by taking time-lapse phase contrast images with an IncuCytePlus microscope at 95 h after transfection (n = 3). Values are mean +/− s.e.m.; statistically significant changes are shown with asterisks (*p<0.05, **p<0.01, ***p<0.001).

**Figure 3:**
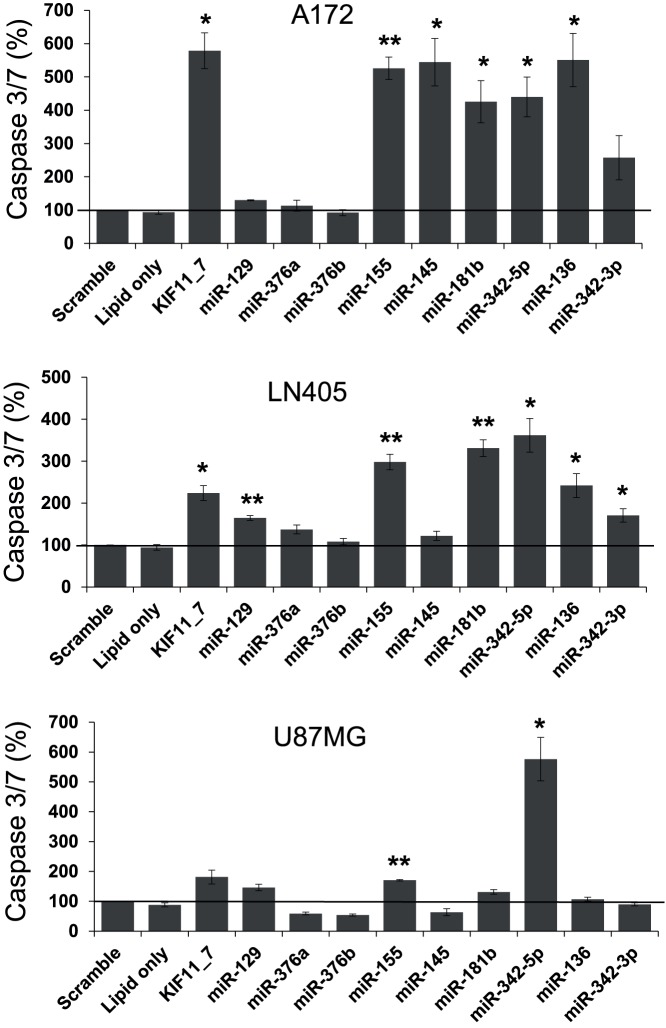
Validation of the precursor hit miRNAs that reduced proliferation with the Caspase 3/7 Glo assay 72 h after transfection (n = 3 biological, 4 technical repeats). Values are mean +/− s.e.m.; statistically significant changes are shown with asterisks (*p<0.05, **p<0.01, ***p<0.001).

Nine miRNA precursor molecules (hsa-miR-129, -136, -145, -155, -181b, -342-5p, -342-3p, -376a/b) were selected for additional validation experiments based on the functional and expression data. The nine miRNAs had an antiproliferative effect in several glioma cancer cell lines, and were less effective in the control cell line SVGp12. First, the functional effects were validated using the CellTiter-Glo assay (Figure 2A). Cell proliferation was also monitored by taking time-lapse phase contrast images with the IncuCytePlus microscope (Essen Instruments) that automatically quantitated cell confluency (Figure 2B). Introducing the AllStars Cell Death Control siRNA or KIF11_7 control siRNAs into the A712, LN405, and U87MG cell lines caused a significant reduction in cell proliferation as determined via ATP quantity. Interestingly, overexpression of miRNA precursors hsa-miR-155, -145, -181b, -136, -129, and -376a/b caused significant reduction in cell proliferation in the A172 and LN405 cell lines. Most of these same miRNA precursors were tested and caused reduction in cell confluency of the A172 and LN405 cell lines, except miR-145, which had a significant effect in time-lapse phase contrast microscopy only in the A172 cell line (Figure 2). The U87MG glioblastoma cell line was less sensitive to the introduction of the nine miRNAs; only hsa-miR-129, -376a, -376b, and -342-5p reduced cell proliferation significantly in the CellTiter-Glo assay, and of these miRNAs, hsa-miR-342-5p significantly reduced cell confluency. The Caspase-Glo 3/7 assay showed miRNA precursors that increase apoptosis when overexpressed in glioma cells (Figure 3). In the A172 cell line, miRNA precursors miR-155, -145, -181b, -342-5p, and -136 caused a clear increase of 400% to 600% in caspase-3 and -7 activity compared to the scrambled siRNA transfected cells. Many of the same miRNA precursors also increased apoptosis in the LN405 cell line, including miR-155, -181b, -342-5p, and -136. Again, the U87MG cell line was less sensitive to the nine miRNAs; only miR-155 and -342-5p caused a significant increase in apoptosis in the U87MG cell line (Figure 3, lowest panel). Lower sensitivity of the U87MG cell line could be caused by lower transfection efficiency as KIF11 cell death controls also showed a smaller effect in the proliferation and apoptosis assays. The cell cycle was analyzed to understand more of the cell proliferation effects in addition to apoptosis. The clearest effects were observed for miR-145, which caused an S phase block to the cell cycle in the A172 cell line and a similar effect in the LN405 cell line although this was not significant (Figure S2). MiR-155 also caused detraction in the cell cycle in the LN405 and U87MG cell lines; however, this effect was not consistent and was more cell line specific.

### Expression of the Proliferation Reducing miRNAs in Clinical GBM Samples

**Figure 4:**
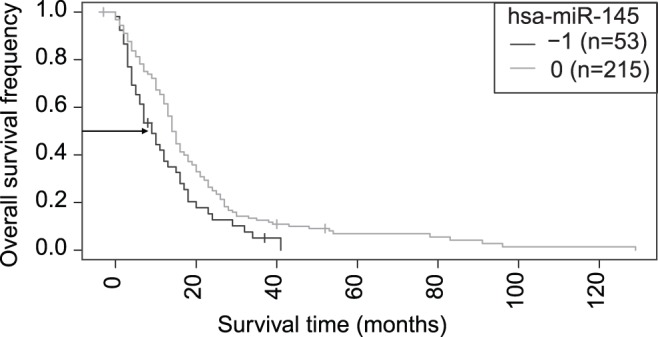
Kaplan-Meier analysis of the hsa-miR-145 miRNA in clinical TCGA GBM samples (n = 268 in total). Significant survival difference with p<0.005 is seen between the hsa-miR-145 low expression group (group denoted with −1, n = 53) who have poor survival and the high or normal expression group (denoted with 0, n = 215) with a threefold change threshold between patient groups.

Next, we wanted to know how these functionally interesting miRNAs were expressed in clinical glioblastoma tumor samples. For eight of the miRNAs (hsa-miR-129,-136, -145, -155, -181b, -342, -376a/b), expression array data were available from the Cancer Genome Atlas (TCGA) project data [14]-[16]. The TCGA miRNA data with 308 GBM patient samples and nine normal samples were analyzed with the Anduril data integration tool [12]. Six miRNAs (hsa-miR-136, -145, -155, -376a, -342, and -129) had significantly different expression values when compared to normal brain tissue (Table 1). All the other miRNAs were expressed at lower levels in GBM than in the normal brain except miR-155, which was expressed almost twofold higher in the GBM samples. The high expression of miR-155 was also seen in our cell lines. Low expression of hsa-miR-145 in GBM tumors was significantly associated with poor survival based on a Kaplan-Meier analysis (Figure 4). The mean survival time for patients with high or low levels (high level: 0 group, low level: –1 group) of hsa-miR-145 was 14.9 months and 10.7 months, respectively. Higher hsa-miR-145 expression in GBM tumors yielded significantly longer survival (p-value<0.005, fold-change threshold = 3, *n* = 53 and *n* = 215 in groups) in a small subset of patients; the longest survival time was 120 months, whereas in the miR-145 low expression group, the longest survival time was only 40 months.

**Table 1:** Significant differences in miRNA expression of the functionally relevant miRNAs in clinical GBM tumor samples (n = 308) compared to normal brain (n = 9) from the TCGA GBM dataset as analyzed with the Anduril tool [12].

miRNA	Expressionp-value	Expression fold change (log2)	Survivalp-value
hsa-miR-136	7.52E-14	−1.69	NS
hsa-miR-145	5.88E-04	−1.04	0.005
hsa-miR-155	1.18E-21	1.94	NS
hsa-miR-181b	5.44E-02	−0.22	NS
hsa-miR-342	4.35E-10	−1.25	NS
hsa-miR-129	1.29E-16	−3.39	NS
hsa-miR-376a	4.35E-07	−0.63	NS
hsa-miR-376b	7.37E-02	0.07	NS

Survival p-value was calculated from miRNA expression data with Kaplan-Meier analysis. Expression and survival p-values are considered significant if the p-value is less than p<5.00E–02. NS not significant.

### Several Putative Targets of the Proliferation Reducing miRNAs in GBMs

**Figure 5:**
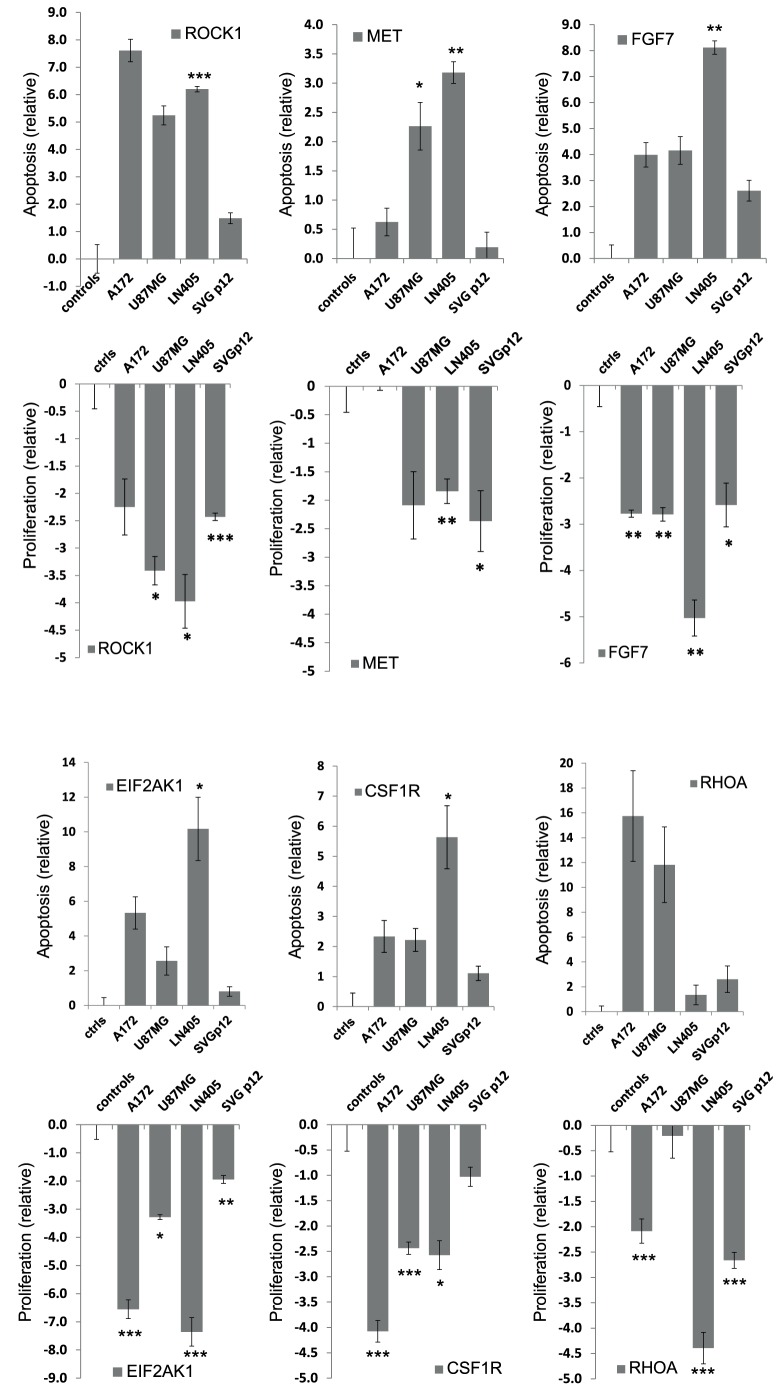
Silencing of selected miRNA target genes in four cell lines to validate results from the glioma siRNA screening: functional assays showing increased apoptosis (ApoOne, Promega) or inhibition of cell proliferation (CellTiter-Glo, Promega) relative to control wells normalized to zero (negative control siRNA or lipid only). *MET*, *CSF1R*, *FGF7*, and *RHOA* are previously experimentally observed mir-155 targets. *ROCK1* and *EIF2AK1* are highly predicted miRNA targets of mir-145 from Target Scan (see Table 3). Values are mean +/− s.e.m., loess normalized log_2_ data from secondary validation screening (n = 3–4). The p-values are two-sided and are compared to the negative control wells. Statistically significant changes are shown with asterisks (*p<0.05, **p<0.01, ***p<0.001).

Each miRNA can target hundreds to thousands of different target genes by silencing them indirectly at the transcriptional and translational levels. Only a few validated miRNA–target relations are currently known, and several programs predict target genes for miRNAs based on the short miRNA seed sequence. Instead of focusing on a single miRNA target, we wanted to study the potential target genes more systematically. The miRNAs that reduced proliferation were analyzed with the IPA program. The predicted and experimentally proven targets of each miRNA that reduced proliferation provided by the IPA program are shown in Table 2. In total, 6,458 target genes were predicted by the IPA program for the eight miRNAs that reduced proliferation found in our study available in IPA, ranging from 448 genes for miR-342-5p to 2,149 genes for the miR-129 cluster. To filter out the most relevant miRNA target genes, we combined our existing high-throughput siRNA screening cell proliferation data (not shown in their entirety) with the miRNA target predictions and filtered in genes that cause reduced proliferation or increased apoptosis when silenced with siRNA transfection in these same cell lines (Table 3). The 10 genes shown in Table 3 were found to cause either reduced proliferation or increased apoptosis in glioma cell lines with two or more siRNAs or in two to three cell lines. Six miRNA target genes are presented here with a reduced proliferation or increased apoptosis phenotype in any of the four cell lines (Figure 5, Table 3). The hsa-miR-155 target genes *MET*, *RHOA*, *CSF1R*, and *FGF7* have been previously experimentally validated as miR-155 targets in other cell line models [17]–[21]. Statistically significant changes are shown for each cell line compared to the control wells (mean of scrambled siRNAs and lipid-only wells normalized to zero). The control cell line SVGp12 generally shows none or smaller proliferation and apoptosis effects with these siRNA transfections compared to the three glioma cell lines (Figure 5). This is in line with the miRNA proliferation effects that were more pronounced with the glioma cell lines than with the control astrocyte cell line.

**Table 2:** Predicted targets of the eight microRNAs (in total 6,458 mRNAs, from IPA).

miRNA	Number of targeted mRNAs
miR-342-5p/miR-4664-5p	448
miR-342-3p	799
miR-129-5p	2149
miR-376a-3p	917
miR-136-5p/miR-136	937
miR-155-5p	983
miR-145-5p/miR-5195-3p/miR-145	1216
miR-181a-5p/miR-181b-5p/miR-181a	1817
Predicted mRNA targets for 8 miRNAs	6458

**Table 3:** Predicted miRNA target genes with proliferation-decreasing or apoptosis-inducing phenotype in siRNA screenings of the glioma cell lines.

miRNA	Source	Confidence	Target gene	Entrez Gene Name	Hit type
hsa-miR-155	TarBase	Experimentally Observed	MET	met proto-oncogene (hepatocyte growth factor receptor)	1
hsa-miR-155	miRecords	Experimentally Observed	RHOA	ras homolog gene family, member A	1
hsa-miR-155	Ingenuity Expert Findings,TargetScan Human	Experimentally Observed	CSF1R	colony stimulating factor 1 receptor	1
hsa-miR-181b	TargetScan Human	High (predicted)	PRKCE	protein kinase C, epsilon	1
hsa-miR-342-5p	TargetScan Human	Moderate (predicted)	MARK2	MAP/microtubule affinity-regulating kinase 2	1
hsa-miR-155	TargetScan Human	High (predicted)	SALL1	sal-like 1 (Drosophila)	1
hsa-miR-155	Ingenuity Expert Findings,TargetScan Human,miRecords	Experimentally Observed	FGF7	fibroblast growth factor 7	1
hsa-miR-145	TargetScan Human	High (predicted)	ROCK1	Rho-associated, coiled-coil containing protein kinase 1	2
hsa-miR-145	TargetScan Human	High (predicted)	CAMK1D	calcium/calmodulin-dependent protein kinase ID	2
hsa-miR-145	TargetScan Human	High (predicted)	EIF2AK1	eukaryotic translation initiation factor 2-alpha kinase 1	2
hsa-miR-181b	TargetScan Human	Moderate (predicted)	EIF2AK1		
hsa-miR-342-5p	TargetScan Human	Moderate (predicted)	EIF2AK1		

Hit type column: 1 = validated with 2 or more siRNAs, 2 = validated in 2–3 cell lines.

The expression of these putative miRNA target genes was also analyzed in the clinical GBM samples from the TCGA data. Five of the 10 genes identified in Table 3 (*CSF1R*, *ROCK1*, *RHOA*, *EIF2AK1*, and *SALL1)* were significantly overexpressed in the GBM data compared to the normal brain in the TCGA cohort [12] following the logic of underexpressed miRNA and an overexpressed target gene. *FGF7* was not significantly differentially expressed, and *MET*, *CAMK1D*, *MARK2*, and *PRKCE* were significantly underexpressed (Table 4). MiR-155 was overexpessed and has been experimentally proven to target *MET* [17], [18], and *MET* was underexpressed in the GBM TCGA cohort following the logic of the overexpressed miRNA and underexpressed target gene. Low *MET* expression was linked to longer survival (*n* = 225) in the TCGA data analyzed with Anduril (Figure S3).

**Table 4:** Significant differences in mRNA expression of the functionally relevant miRNA target genes in the clinical GBM tumor samples (n = 308) compared to normal brain (n = 9) from the TCGA GBM dataset as analyzed with the Anduril tool [12].

	Gene expression			Transcript expression		
Gene	Fold change	p-value	Survivalp-value	Min.	Max.	Survivalp-value
MET	0.388	7.03e-9	0.0964	0.297	0.532	0.0368
CSF1R	1.41	0.00528	NA	1.34	2.02	NA
FGF7	0.743	0.392	NA	0.769	0.769	NA
ROCK1	1.79	8.57e-5	NA	1.86	1.86	NA
RHOA	1.47	2.43e-5	NA	1.44	1.59	NA
EIF2AK1	1.47	4.67e-5	NA	1.47	1.53	NA
SALL1	2.15	2.58e-8	0.883	2.01	2.39	0.225
CAMK1D	0.176	7.60e-9	NA	0.140	0.307	0.00533
MARK2	0.602	7.24e-6	NA	NA	NA	NA
PRKCE	0.165	8.83e-12	NA	0.162	0.509	NA

The gene expression measures the expression of the exons linked with the particular gene in Ensembl. Expression values are considered significant if the p-value is less than p<5.00E–02. Transcript expressions are calculated from the exon expression with linear algebra-based method discussed in MEAP [13]. Minimum transcript expression denotes minimum splice variant expression and maximum transcript expression denotes maximum splice variant expression. Survival p-values were calculated from the gene expression data with Kaplan-Meier analysis. NA, Not Available.

## Discussion

Since microRNAs were discovered, there have been several publications of miRNA expression in various cancers. Generally, microRNAs are mainly down-regulated in cancers, as is the case with miR-128, miR-181a, miR-181b, and miR-181c in glioblastomas [7]. Only a few miRNAs, such as miR-221 and miR-21, are strongly up-regulated in GBM and grade II–IV astrocytic tumors [8]. In our study, we systematically analyzed the functions of miRNAs by overexpressing precursor miRNAs in glioma cell lines and assaying the proliferation and apoptosis phenotypes. The hypothesis behind our approach was to find miRNAs that would be down-regulated at the expression level in gliomas and whose function was tumor suppressor–like when overexpressed in this cancer type. We found 24 potential precursor miRNA molecules that reduced proliferation in the primary screening, and nine of these miRNAs were validated in more detail for their effects on reducing proliferation or inducing apoptosis. According to the CellTiter-Glo assay, overexpression of seven miRNA precursors (hsa-miR-155, -145, -181b, -136, -129, and -376a/b) caused significant reduction in cell proliferation in the A172 and LN405 cell lines, whereas four miRNA precursors (hsa-miR-129, -376a, -376b, and -342-5p) significantly reduced cell proliferation in the U87MG cell line. The lower response rate in the U87MG cell line to miRNA overexpression could be caused by lower transfection efficiency. Five of the miRNA precursors (hsa-miR-155, -145, -181b, -342-5p, and -136) induced apoptosis via caspase-3 and -7 activation. Five miRNAs, hsa-miR-136, -145, -376a, -342, and -129, showed significantly lower expression values in the GBM patient tissue samples compared to normal brain tissue.

We found that hsa-miR-181b functions as a tumor suppressor and overexpression inhibited growth and induced apoptosis. Similar results have been found previously, as hsa-miR-181a and especially hsa-miR-181b mature miRNAs have been shown to be expressed at low levels in GBM and miR-181b in astrocytomas compared to the normal brain [8], [22] as well as function as tumor suppressors and inhibit invasion of glioma cells [23]. These findings are in line with our findings for miR-181b. In addition, low miR-181b expression levels have been found to correlate with poor survival in patients with astrocytomas compared to patients with high miR-181b expression levels (p-value<0.039, [22]). This survival effect for miR-181b was not seen in the TCGA GBM cohort.

MiR-342-5p is predicted to target *EIF2AK1*, which we found was a proliferation-reducing hit when silenced in the GBM cell lines. Interestingly, *EIF2AK1* is a predicted target of three proliferation-connected miRNAs in this study, and *EIF2AK1* acts at the level of translation initiation to down-regulate protein synthesis in response to stress [24] and its overexpression can cause loss of cell proliferation [25]. MiR-342 is an important mediator of tamoxifen response in breast tumor cell lines and breast cancer patients; restoring miR-342 expression sensitized breast cancer cells to tamoxifen-induced apoptosis with a dramatic reduction in cell growth [26].

Hsa-miR-145 was expressed at significantly lower levels in GBM patient tissue samples and cell lines when compared to normal brain tissue, and low expression of miR-145 in GBM tumors was significantly associated with poor survival based on a Kaplan-Meier analysis of the TCGA GBM data. Overexpression of the miR-145 precursor reduced cell proliferation in the A172 and LN405 cell lines and increased apoptosis in the A172 cell line. Previously, the pluripotency factors *OCT4*, *SOX2*, and *KLF4* were shown to be direct targets of miR-145. Increased miR-145 expression inhibits self-renewal, represses expression of pluripotency genes, and induces lineage-restricted differentiation in human embryonic stem cells [27]. In glioblastoma multiforme, *SOX2* down-regulates miR-145, and both are probably involved in a double-negative feedback loop in maintaining the stemness of glioma stem cells [27], [28]. The predicted targets of miR-145, *ROCK1* and *EIF2AK1*, were proliferation-reducing siRNA hits in our study resembling the miR-145 proliferation effect and thus proving these experimentally as novel putative miRNA targets. Expression of *ROCK1* and *EIF2AK1* was up-regulated in the clinical GBM TCGA samples conforming to down-regulated miR-145. Interestingly, Rho/ROCK signaling has been previously shown to be involved in GBM cell migration and proliferation via use of ROCK inhibitor Y-27632 or siRNA silencing [29].

Unlike the other underexpressed tumor suppressor miRNAs found in this study, microRNA-155 is a known oncomir in many cancers, and is thought to regulate multiple genes associated with cancer cell proliferation, apoptosis, and invasiveness [30], [31]. Elevated miR-155 levels were found in the TCGA GBM data and in other datasets such as primary and secondary glioblastoma tissues, glioblastoma primary cultures [32], and the cell lines in our study. MiR-155 has been previously shown to target many genes, e.g., *MET* [17], [18], *FGF7* [19], *RHOA* [20], and *CSF1R* [21] all whose silencing reduced the GBM cell line proliferation in our study. As *RHOA*, *CSF1R*, and miR-155 were all overexpressed in clinical GBM samples compared to normal brain tissue, they do not form a logical reciprocal miRNA - target gene pair, and therefore also other mechanisms not yet understood must be involved here. However, *MET* expression was low in the clinical GBM samples, and miR-155, which has been shown to target *MET*, was overexpressed, thus establishing a logical association between the overexpressed miRNA and underexpressed target pair. Low *MET* expression was linked to longer survival in the majority of the clinical GBM samples (*n* = 225), and in only a small number of patients (*n* = 22) was high *MET* oncogene transcript expression linked to poor survival. The hypothesis behind our approach was to find miRNAs that would be down-regulated at the expression level in gliomas and whose function would be tumor suppressor–like when overexpressed in this cancer type. We were successful in discovering nine novel functional miRNAs in the GBM cell lines and, with the indirect functional siRNA validation approach showed six putative novel target genes for these miRNAs. Four of the six target genes, *MET*, *RHOA*, *CSF1R*, and *FGF7*, have been previously shown to be direct targets of these miRNAs, and the two remaining, *ROCK1* and *EIF2AK1*, are predicted miRNA targets. Thus, although these target genes were not shown as direct target genes here, we believe that combining the miRNA and siRNA screening data can provide novel and more systematic understanding of miRNA biology and cancer biology in general than focusing on determining a single direct miRNA target. The TCGA GBM clinical miRNA and gene expression data confirmed our cell line findings, showing the relevance of the traditional cell line models recently shown in two big cell line–based studies [33], [34]. Functional miRNA screening studies will provide vast amounts of information on the unknown microRNA functions and when integrated with siRNA functional studies will uncover the general vulnerabilities of cancer cell pathways.

## Supporting Information

Figure S1 **Hierarchical clustering of repeated hits from the miRNA pre-miR functional proliferation secondary screening using the Euclidean method in MeV 4.8 (Loess log normalized log_2_ data)** [**10]**, [11] **.** Each screening was performed with three to four biological replicates. Arrows denote miRNAs that were selected for further validation.
(PDF)

Figure S2 **Cell cycle analysis after miRNA precursor overexpression using the Click-it Edu Alexa 647 nm fluor HCS assay kit (Invitrogen Carlsbad, CA, USA) and Hoechst 33342 stain for the A172, LN405, and U87MG cells.** Each assay was repeated three times with a 48 h time point after miRNA transfection. Values are mean +/− s.e.m.; statistically significant changes are shown with asterisks (*p-value<0.05, **p-value<0.01, ***p-value<0.001).
(PDF)

Figure S3 **Kaplan-Meier analysis of the** ***MET*** **oncogene in the clinical GBM samples (n = 382 in total).** Significant survival difference with p<0.0368 was seen between the *MET* low expression group (group denoted with –1, blue line, n = 225) with better survival and the high expression group (denoted with 1, red line, n = 22) with a twofold change threshold between the patient groups. The normal average expression group is denoted with a green line (0 group, n = 135).
(PDF)

Table S1 An Excel file of screening data from miRNA pre-miR libraries in glioma cell lines. **Sheet 1)** Data from the human pre-miR miRNA Precursor library v2 (Ambion Inc., 319 molecules) screening. Data have been normalized for row and column effects. **Sheet 2)** Data from the human miRIDIAN microRNA Mimic Libraries v10.1 (Dharmacon, Lafayette, 819 molecules) screening. Data are presented as Loess log normalized log_2_ data.
(XLSX)
