# Supplementary figures and images for: Functional Profiling of Precursor MicroRNAs Identifies MicroRNAs Essential for Glioma Proliferation
Source: PLoS One. 2013 Apr 5;8(4):e60930. doi: 10.1371/journal.pone.0060930 (PMC3618426; doi:10.1371/journal.pone.0060930)

Figure S1. Haapa-Paananen et al.

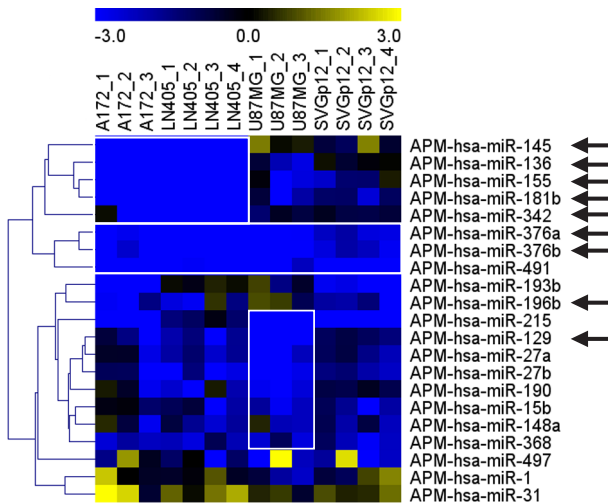

Supplement: Figure S1 — Hierarchical clustering of repeated hits from the miRNA pre-miR functional proliferation secondary screening using the Euclidean method in MeV 4.8 (Loess log normalized log2 data) [10] , [11] . Each screening was performed with three to four biological replicates. Arrows denote miRNAs that were selected for further validation. (PDF) [file pone.0060930.s001.pdf]

Figure S2. Haapa-Paananen et al.

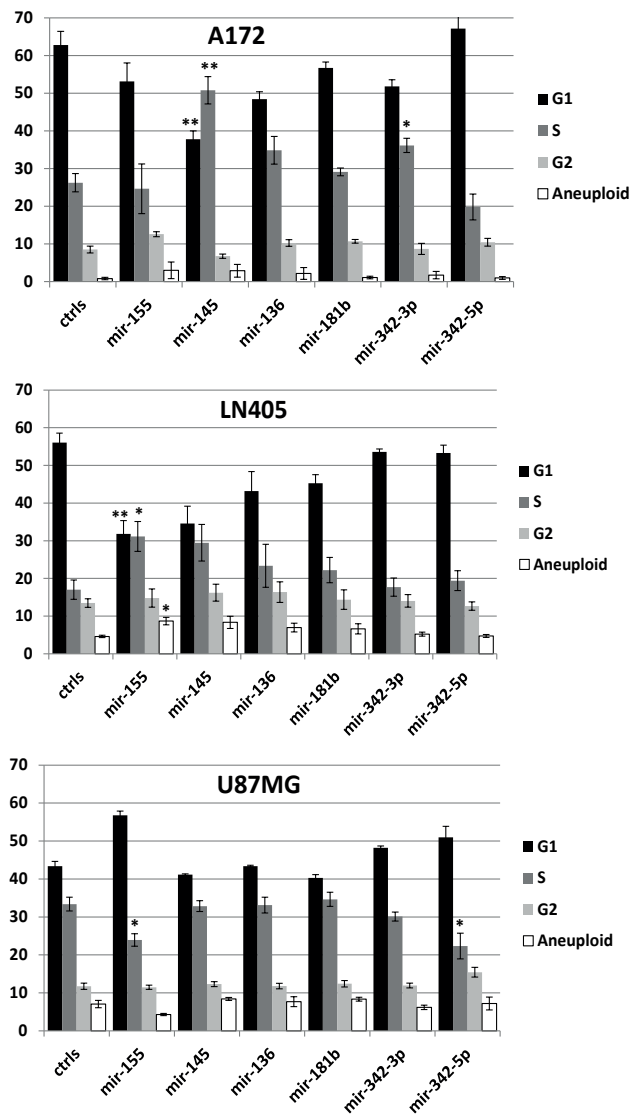

Supplement: Figure S2 — Cell cycle analysis after miRNA precursor overexpression using the Click-it Edu Alexa 647 nm fluor HCS assay kit (Invitrogen Carlsbad, CA, USA) and Hoechst 33342 stain for the A172, LN405, and U87MG cells. Each assay was repeated three times with a 48 h time point after miRNA transfection. Values are mean +/− s.e.m.; statistically significant changes are shown with asterisks (*p-value<0.05, **p-value<0.01, ***p-value<0.001). (PDF) [file pone.0060930.s002.pdf]

Figure S3. Haapa-Paananen et al.

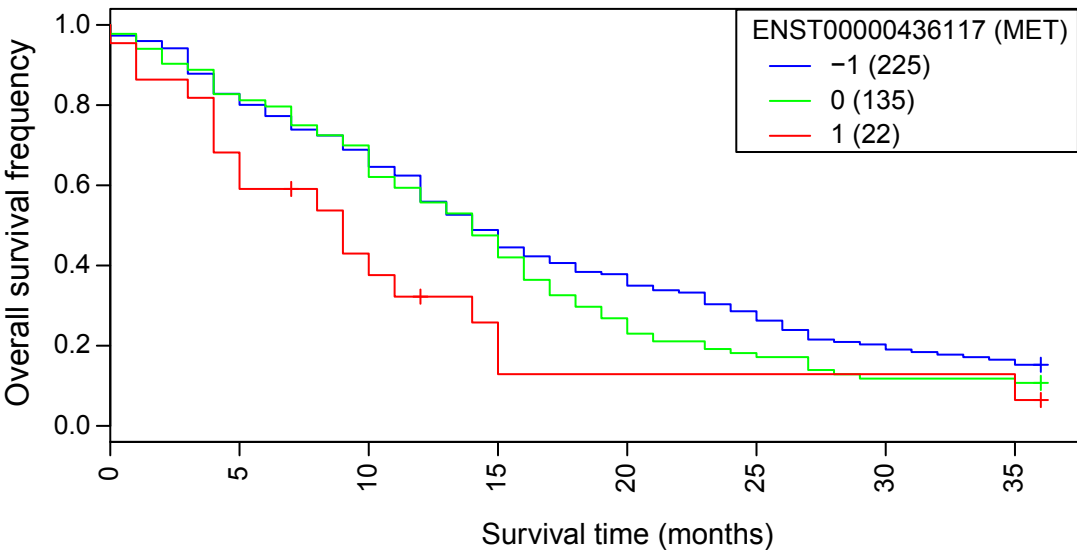

Supplement: Figure S3 — Kaplan-Meier analysis of the MET oncogene in the clinical GBM samples (n = 382 in total). Significant survival difference with p<0.0368 was seen between the MET low expression group (group denoted with –1, blue line, n = 225) with better survival and the high expression group (denoted with 1, red line, n = 22) with a twofold change threshold between the patient groups. The normal average expression group is denoted with a green line (0 group, n = 135). (PDF) [file pone.0060930.s003.pdf]
